# Propeller-Like All-Fused Perylene Diimide Based Electron Acceptors With Chalcogen Linkage for Efficient Polymer Solar Cells

**DOI:** 10.3389/fchem.2020.00350

**Published:** 2020-04-29

**Authors:** Ying Li, Yufei Gong, Yongjie Che, Xiaopeng Xu, Liyang Yu, Qiang Peng

**Affiliations:** Key Laboratory of Green Chemistry and Technology of Ministry of Education, College of Chemistry and State Key Laboratory of Polymer Materials Engineering, Sichuan University, Chengdu, China

**Keywords:** polymer solar cells, non-fullerene acceptor, perylene diimide, propeller-like, all fused molecular structure

## Abstract

Perylene diimide (PDI) is a widely explored chromophore for constructing non-fullerene acceptors (NFAs) for polymer solar cells (PSCs). The advantage of using PDI derivatives lies in the readily availability of PDI unit which largely reduces the synthesis cost and improves material stability. Indeed, the recent development of high performance NFAs shed light on the feasibility of the commercialization, but the complex synthesis and poor stability of the top performing NFAs cast a shadow on this bright future. Our previous work has demonstrated a propeller-like structure with three PDIs lined to a benzene center core with a C-C bond which prevented the PDIs to aggregate into undesired large crystals. In this work, we designed and synthesized three new propeller-like PDI derivatives with extra chalcogen linkages between the PDIs and the center core to form all-fused rigid structures. These molecules showed more suitable absorption range than that of their unfused counterparts when blend with donor polymer PTB7-Th. Comparing between the molecules with extra oxygen, sulfur or selenium linkages, the sulfur-based BTT-PDI outperformed the others due to its higher photon absorption and charge transport abilities. This work demonstrated the great potential of PDI derivatives for PSC applications and explored the influences of linkage type on the fused PDI derivatives, which provided a useful tuning knob for molecular design of PDI-based NFAs in the future.

## Introduction

Polymer solar cell (PSC) is widely considered as a viable alternative for solar energy harvesting for its relatively low manufacturing cost and intrinsic characteristics such as light weight and flexibility (Inganäs, 2018). The recent development of non-fullerenes acceptor (NFA) molecules has promoted this technology by delivering largely increased power conversion efficiency (PCE) of the PSCs (Cheng et al., 2018; Hou et al., 2018; Yan et al., 2018; Zhang G. et al., 2018; Xu et al., 2019a). Top performing devices using NFAs have exceed 16% in PCE, which demonstrate a bright future for this technology maturing into commercialization (Cui et al., 2019; Sun et al., 2019; Xu et al., 2019b, 2020; Yan et al., 2019; Yu et al., 2019).

On the other hand of the fast rising top PCEs lies a reality that the used NFAs are often with complex and costly synthesis routes. The high synthesis cost, which impaired the full exploration of the low-cost advantage of PSCs, was mainly raised because each of the component chromophores needed to be bottom-up synthesized. Hence, the utilization of readily available chromophores has drawn increasing attention as the potential of commercialization emerges. PDI and its derivatives are long explored candidates for NFAs (Duan et al., 2017a; Hou et al., 2018; Yan et al., 2018; Zhang G. et al., 2018; Zhang J. et al., 2018; Genene et al., 2019), because the PDI chromophore is relatively inexpensive and with high electron mobilities. However, the flat PDI molecules are well-known for strong aggregation (Hartnett et al., 2014). Large crystals of PDI derivatives delivered outstanding performances for N-type transistors (Zhan et al., 2011) but deviated the PSC nanostructure far from the optimized phase separation scale, which limits the PCE (Zhan et al., 2011).

Connecting a few PDI molecules was a method widely explored to disturb the crystallization of PDI derivatives. In comparison with connecting the PDIs heat to tail (Ye et al., 2015; Liang et al., 2016; Eastham et al., 2017), a more widely adopted method was to connect the PDIs at the bay positions. Connecting two or more PDI side by side by either ridged (Zhong et al., 2014, 2015, 2016; Eastham et al., 2017; Sisto et al., 2017; Wang et al., 2020) or flexible (Yan et al., 2013; Zhang et al., 2013; Liu et al., 2016; Meng et al., 2016a; Wu et al., 2018; Kim et al., 2019) linkages at the bay positions have been demonstrated to optimize the nanostructure of PSCs. These linked PDI molecules were found to be twisted with chromophores not positioned in the same plane, which was a cause for the reduction in crystallization tendency. A propeller-like structure connecting three or four PDI molecules with a center core through a flexible linkage have also been demonstrated with success (Lin et al., 2014, 2016; Lee et al., 2016; Duan et al., 2017b; Sun et al., 2017; Bian et al., 2019; Tang et al., 2019; Weng et al., 2019; Zhang et al., 2019; Ding et al., 2020; Wang et al., 2020). Strong steric hindrance effect twisted the molecules sharply and large coplanar angles where found between these PDIs and the center core. The 3D structure largely reduced the aggregation tendency of PDIs and thin films containing these molecules were often found to be amorphous. Fusing the PDIs on the center core can reduce the coplanar angle between them (Meng et al., 2016b; Wang et al., 2019; Wu et al., 2019). Hence fused rigid propeller-like structure were found with both improved intra- and intermolecular charge transport (Meng et al., 2016b; Wang et al., 2019; Wu et al., 2019).

**Scheme 1:**
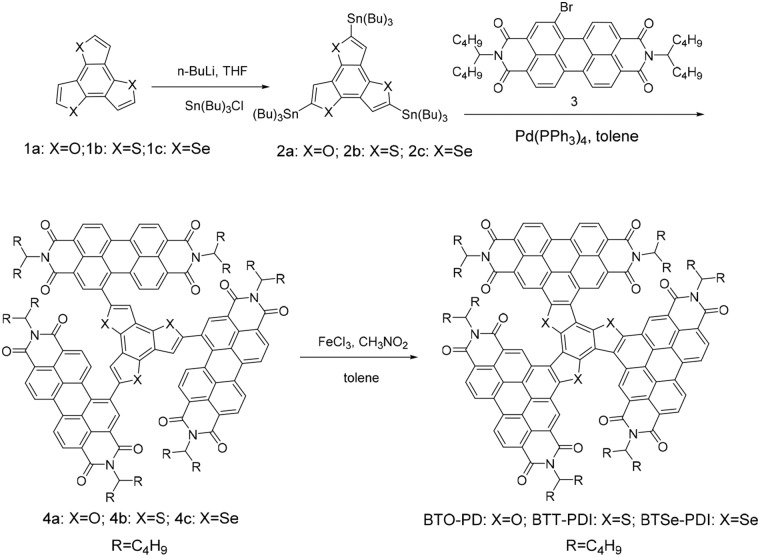
Synthesis routes for the NFAs presented in this work.

Particularly, triperylene hexaimides (TPH) with PDI molecules simply fused together by a benzene ring was a successful example of fused propeller-like structure with three PDI units delivering high PCE (Meng et al., 2016b). The small center core unit dramatized the steric hindrance effect and the molecule was found with an extremely twisted structure. In this work, we elegantly separated the three PDIs of the TPH with an additional planar five-membered heterocyclic ring between the PDIs and the benzene ring at the center. This design was in hope to further extend the conjugation length and reduce the molecule torsion. The choice of the additional five-membered heterocyclic ring was also investigated. Furan (BTO-PDI), thiophene (BTT-PDI), or selenophene (BTSe-PDI) were added between the PDIs and the center benzene ring by introducing additional oxygen, sulfur, or selenium linkages to the flexible linked Ph-PDI molecule we reported previously (Duan et al., 2017b). The chemical structures and the synthesis routes of these three NFAs are presented in Scheme 1. In fact, chalcogens were often used to decorate the bay position of the PDIs to influence their optoelectronic properties (Li et al., 2019, 2020). Differences in size and electron withdrawing ability of the three chalcogen elements shall influence both the molecular conformations, energetic structures, and device performances of these PDIs (Heeney et al., 2007; Chen et al., 2010; Das and Zade, 2010; Jahnke et al., 2013). The molecules developed in this work shows more delocalized and deeper HOMO level than TPH (Meng et al., 2016b) and Ph-DPI (Duan et al., 2017b). The larger band gap caused by the deeper HOMO level formed an improved complimentary absorption of these three new NFAs incorporated with the donor polymer PBT7-Th comparing with TPH and Ph-PDI. The highest performances were observed with BTT-PDI for its high photon absorption and charge transport mobility.

## Results and Discussions

### Thermal Study

**Figure 1:**
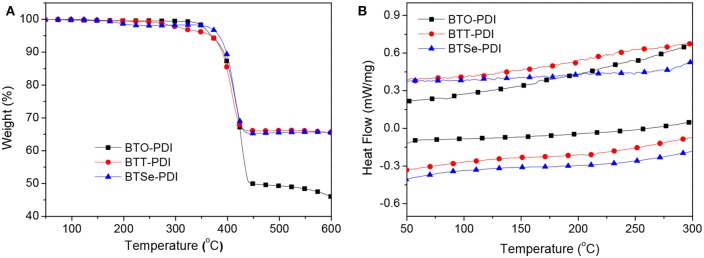
TGA **(A)** and DSC **(B)** thermograms of the three NFAs.

Thermal stabilities of the three NFAs were examined by thermogravimetry analysis (TGA, Figure 1A). All the three materials showed a single weight loss as a step function. The temperatures reaching 5% weight loss were recorded for BTO-PDI, BTT-PDI, and BTSe-PDI at 368, 376, and 381°C, respectively, as the decomposition temperature. Hence, all the three molecules could be considered thermally stable for PSC application. Differential scanning calorimetry (DSC, Figure 1B) scans of the three NFAs were also performed to look for thermal transitions. However, no endo- or exothermal transition was observed between 50 and 300°C for any of the three molecules during heating and cooling at rate of 10°C/min suggesting no melting, phase transformation or other secondary transitions in this temperature range for all the three NFAs.

### Optical Property

**Figure 2:**
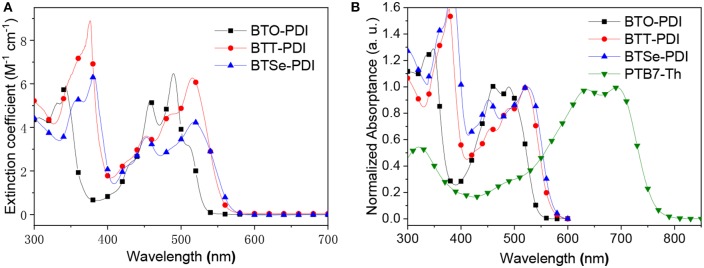
UV-vis absorption spectra of chloroform solution containing the acceptors **(A)** and thin films of the donor and acceptor materials used in this work (**B**, normalized).

UV-vis absorption spectroscopy was conducted on the three NFAs to characterize their photon absorption abilities as well as band structures. Strong absorptions of the three molecules in chloroform solution were found in 300–400 nm region and 420–550 nm region with distinguishable vibronic structures as shown in Figure 2A. The maximum extinction coefficient of the BTO-PDI, BTT-PDI, and BTSe-PDI in 300–400 nm region reached 5.8 × 10^4^ M^−1^ cm^−1^(at 330 nm), 8.9 × 10^4^ M^−1^ cm^−1^ (at 376 nm), 6.6 × 10^4^ M^−1^ cm^−1^ (at 381 nm), respectively. In the 420–550 nm region, maximum extinction coefficient for BTO-PDI, BTT-PDI, and BTSe-PDI were found to be 6.5 × 10^4^ M^−1^ cm^−1^ (at 490 nm), 6.3 × 10^4^ M^−1^ cm^−1^ (at 516 nm), 4.2 × 10^4^ M^−1^ cm^−1^ (at 518 nm), respectively. BTT-PDI absorbed significantly more at the low wavelength region. However, BTO-PDI show slightly higher maximum extinction coefficient than BTT-PDI in the high wavelength region. Additionally, the maximum absorption edge was found to be red shifted as the linking chalcogen getting heavier. This agreed with the observation in most conjugated molecular systems containing chalcogens that heavier chalcogen atoms provides poorer electron withdrawing strength and larger distortion of the five-membered ring both of which favor the extension of conjugation (Heeney et al., 2007; Chen et al., 2010; Das and Zade, 2010; Jahnke et al., 2013). Because the absorption range of BTT-PDI extended to significantly (46 nm) higher wavelengths than BTO-PDI, the BTT-PDI was found with stronger absorption in the high wavelength region as well. In a contrast, the absorption of BTSe-PDI was found to be relatively low in both regions. Absorption spectra of thin films containing neat NFAs and the donor polymer were presented in Figure 2B. The vibronic structures of the absorption spectra for the three molecules were all found to be similar and slightly red shifts comparing with their solutions suggesting that the aggregation of molecules in solid-state was weak. The largest red shift of 15 nm was found for BTT-PDI indicating its strongest inter-molecular aggregation and interaction among the three (Brown et al., 2003; Spano and Silva, 2014). The maximum absorption wavelength (λ_onset_) of the three molecules were used to extract the optical bandgap ($I_{\text{g}}^{\text{opt}}$) using equation $E_{\text{g}}^{\text{opt}}$ = 1,240/λ_onset_. The $E_{\text{g}}^{\text{opt}}$ for BTO-PDI, BTT-PDI, and BTSe-PDI were found at 2.25, 2.20, 2.20 eV, respectively. Compared with the TPH (Meng et al., 2016b) with a simple benzene core or the unfused Ph-PDI (Duan et al., 2017b), the absorption of these three NFAs overlaps less with the absorption of the donor polymer PTB7-Th, suggesting an improved complimentary absorption.

### Electrochemical Study

**Figure 3:**
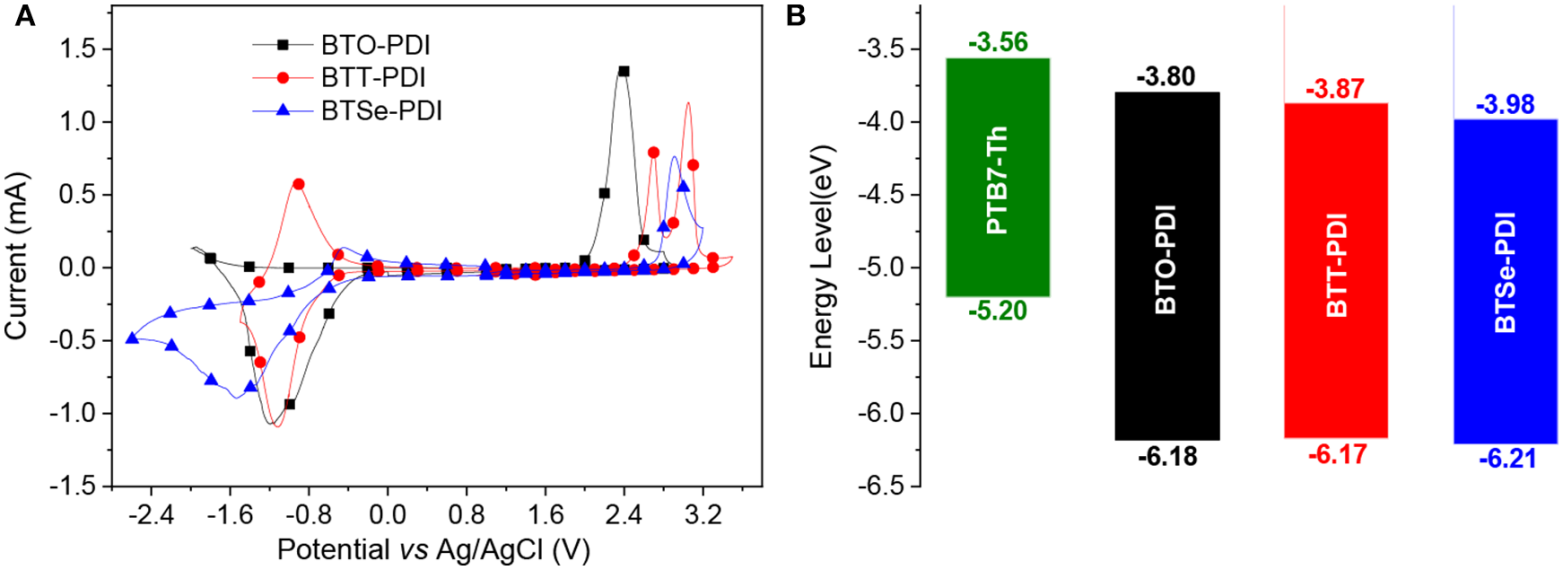
**(A)** Cyclic voltammograms (CV) of the three NFAs, **(B)** energy diagrams of the three NFAs in comparison with the donor polymer PTB7-Th.

Electrochemical analysis was carried out using cyclic voltammetry (CV, Figure 3A) to measure the highest occupied molecular orbital (HOMO) and lowest unoccupied molecular orbital (LUMO) levels of the NFAs. The energy levels of these frontier orbitals were summarized in Table 1 and compared with the donor polymer in Figure 3B. The energy levels of the frontier orbitals (HOMO/LUMO) of BTO-PDI, BTT-PDI, and BTSe-PDI were found at −6.18/−3.80, −6.17/−3.87 and, −6.21/−3.98 eV, with extracted bandgap of 2.38, 2.30, and 2.23 eV, respectively. Deeper LUMO levels were found for the molecules with heavier chalcogens while the HOMO levels were hardly affected leading to a clear reduction in bandgap agreeing with the red shifted UV-vis absorption. A lower LUMO level of the acceptor molecule can lead to reduced open circuit voltage (*V*_oc_) of the PSC devices for molecules with heavier chalcogens linkage. The HOMO level of these NFAs were significantly deeper than that of the TPH (*E*_HOMO_ = 6.02 eV) (Meng et al., 2016b) or the Ph-PDI (*E*_HOMO_ = 6.02 eV) (Duan et al., 2017b) suggesting that it was strongly affected by the linkage type of the PDI units to the center core while the LUMO level remained within proximity.

**Table 1:** Photonic and electrochemical properties of the acceptors.

**Material**	**Solution**		**Film**	**$E_{\text{g}}^{\text{opt}}$ (eV)**	**HOMO (eV)**	**LUMO (eV)**	***E*$E_{\text{g}}^{\text{cv}}$ (eV)**
	**λ_**max**_ (nm)**	**ε (M^**−1**^ cm^**−1**^)**	**λ_**max**_ (nm)**				
BTO-PDI	490	6.5 × 10^4^	348	2.25	−3.80	−6.18	2.38
BTT-PDI	376	8.9 × 10^4^	387	2.20	−3.87	−6.17	2.30
BTSe-PDI	381	6.6 × 10^4^	393	2.20	−3.98	−6.21	2.23

### Molecular Simulations

**Figure 4:**
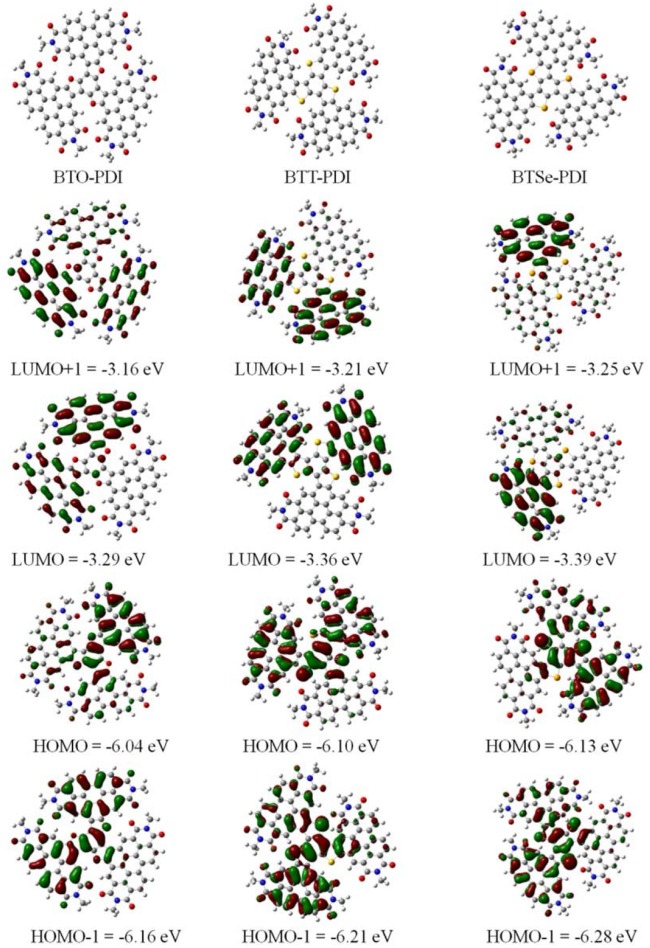
Calculated distribution and energy levels of the frontier orbitals of the three NFAs based on the density function theory (DFT).

To obtain further insight on the molecular conformation and the frontier orbitals, molecular simulation based on density function theory (DFT, B3LYP/6-31G(d) level) were carried out for these three NFAs. The molecular conformations were clearly less twisted comparing with the benzene core TPH (Meng et al., 2016b) and unfused Ph-PDI (Duan et al., 2017b). From the calculated frontier orbital distribution displayed in Figure 4, the HOMO of all three NFAs were found to be largely delocalized over more than one PDI unit while the LUMOs were almost localized to one or two isolated PDI units. Hence, the HOMO levels were more sensitive to the connection between the PDI units and the center core, which explained the more significant deeper HOMO levels of these NFAs than the TPH and the Ph-PDI. The calculated frontier orbital energy levels (HOMO/LUMO) for BTO-PDI, BTT-PDI, and BTSe-PDI were −6.04/−3.29, −6.10/−3.36, −6.13/−3.39 eV, respectively. The increasing trend in the HOMO levels and LUMO levels within an approximate range agreed with optical and CV measurements.

### Photovoltaic Performances

**Figure 5:**
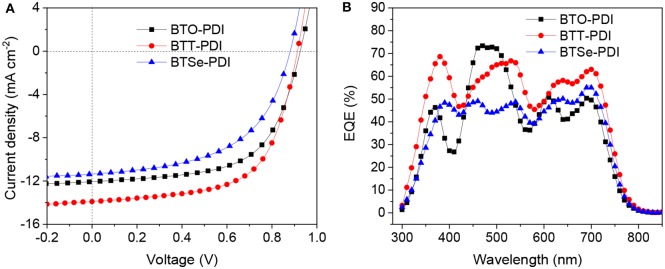
*J*-*V* characteristics **(A)** and EQE responses **(B)** of the top performing PSC devices, the acceptor used in each PSC was noted in the figures.

The PSC devices containing the three NFAs were fabricated in an inverted architecture. The *J*-*V* characteristics of top performing devices were presented in Figure 5A and the extracted figure-of-merits were summarized in Table 2. The *V*_oc_ of devices containing BTO-PDI, BTT-PDI, and BTSe-PDI were found at 0.924, 0.910, and 0.876 V, respectively. The decreasing of *V*_oc_ as the linking chalcogen getting heavier agreed with the decreasing LUMO levels of the NFAs. In a contrast, higher *J*_sc_ and the fill factors (FF) were found for devices containing BTT-PDI (*J*_sc_ = 13.91 mA/cm^2^, FF = 62.22%). BTO-PDI delivered lower *J*_sc_ (12.06 mA/cm^2^) and FF (60.74%) yielding an overall PCE of 6.77% which was lower than that of BTT-PDI (PCE = 7.87%). All figure-of-merits of photovoltaic performances of devices containing BTSe-PDI were lower than those of the other two NFAs with *J*_sc_ = 11.35 mA/cm^2^, FF = 54.17% and PCE = 5.39%.

**Table 2:** Photovoltaic performance of the PSC devices.

**Active layer**	$V_{\text{oc}}^{\text{a}}$ **(V)**	***J*_**sc**_ (mA/cm^**2**^)**	**FF (%)**	**PCE (%)**
PTB7-Th:BTO-PDI	0.919 ± 0.006 (0.924)	11.88 ± 0.31 (12.06)	60.01 ± 0.95 (60.74)	6.55 ± 0.35 (6.77)
PTB7-Th:BTT-PDI	0.907 ± 0.005 (0.910)	13.79 ± 0.35 (13.91)	61.65 ± 0.76 (62.22)	7.71 ± 0.21 (7.87)
PTB7-Th:BTSe-PDI	0.873 ± 0.005 (0.876)	11.11 ± 0.32 (11.35)	52.97 ± 1.44 (54.17)	5.14 ± 0.36 (5.39)

*^a^Averaged parameters were calculated from 10 devices*.

External quantum efficiencies (EQE) were measured for these devices to obtain understanding on the *J*_sc_ differences. The EQE responses of these PSC devices were displayed in Figure 5B. In the 300–400 nm region, BTT-PDI generated high EQE up to 70% while the other two materials delivered EQE under 50% within this range. A sharp drop of EQE around 400 nm for BTO-PDI was noticed, which agreed with its low extinction coefficient at this wavelength. In a contrast, BTO-PDI delivered high maximum EQE in the 420–550 nm region due to its relatively high extinction coefficient in this range. Above 550 nm, the EQE responses were mainly generated from the photons absorbed by the donor polymer PTB7-Th. Interestingly, BTT-PDI provided higher EQE response than the other two NFAs. Hence, there are additional causes for the differences in *J*_sc_ other than the photon absorption ability of the NFAs. The total integrated *J*_EQE_ were found to be similar to the *J*_sc_ of the devices.

### Charge Carrier Mobilities

**Figure 6:**
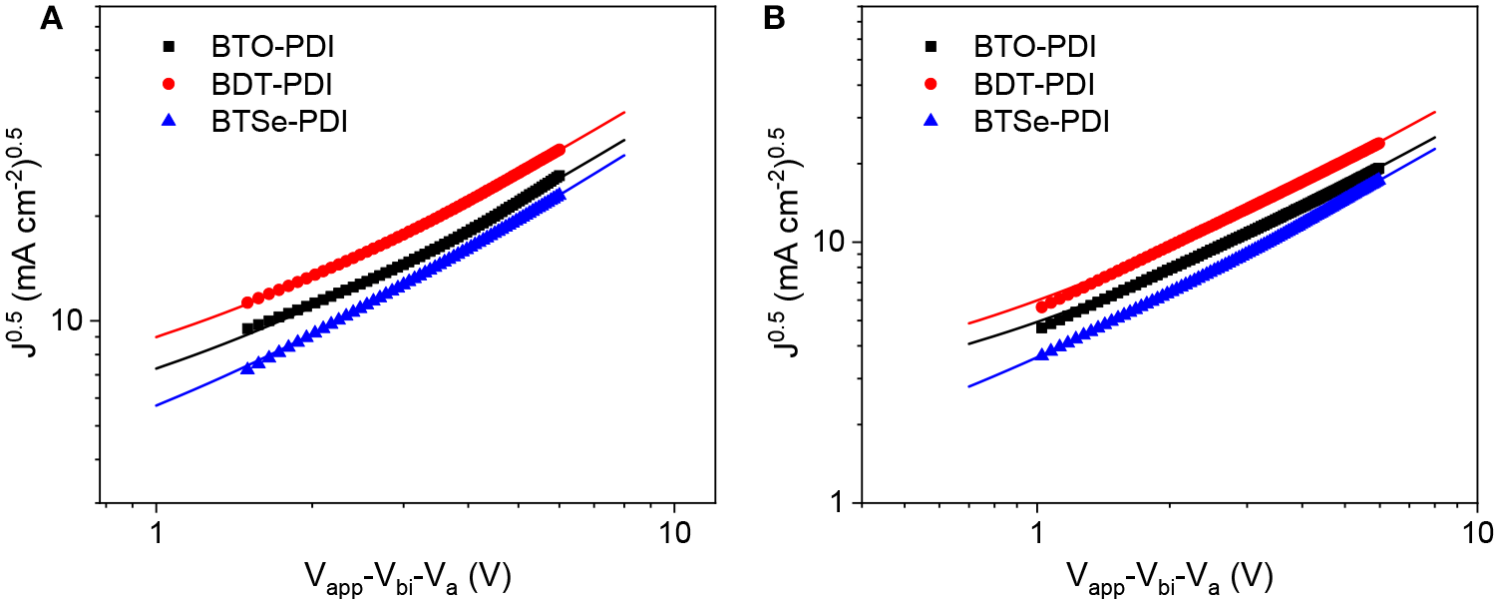
SCLC measurements for extracting hole **(A)** and electron **(B)** transport mobility for thin films containing PTB7-Th in blend with each of the three NF ninety-nine noted in the figures).

The charge carrier mobilities of the PTB7-Th/acceptor blends were measured using space charge limited conductivity (SCLC, Figure 6) method (Malliaras et al., 1998) to further understand the origin for the differences in *J*_sc_ and FF. The SCLC measurements were conducted in hole only and electron only devices to obtain hole mobility (μ_h_) and electron mobility (μ_e_) separately. The extracted charge carrier mobilities (μ_h_/μ_e_)of PTB7-Th in blend with BTO-PDI, BTT-PDI, and BTSe-PDI were found to be 4.51 × 10^−4^/2.79 × 10^−4^, 6.43 × 10^−4^/4.44 × 10^−4^, and 3.98 × 10^−4^/2.49 × 10^−4^ cm^2^ V^−1^ s^−1^ with μ_h_/μ_e_ ratio of 1.62, 1.45, and 1.60, respectively. The significantly higher μ_e_ from the BTT-PDI agrees with its strongest red shift between its thin film and solution. The higher μ_e_ and the relatively more balanced μ_h_/μ_e_ ratio could lead to a more efficient charge transferring process and higher *J*_sc_ and FF (Andersson et al., 2011).

### Crystallinity and Blend Morphology

**Figure 7:**
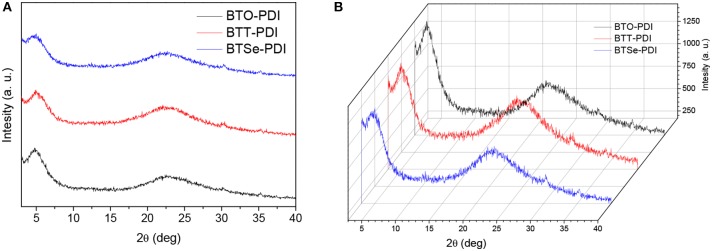
Strength ratio **(A)** and Watrefall **(B)** XRD diffractograms of thin films containing PTB7-Th in blend with each of the three NFAs (noted in the figures).

**Figure 8:**
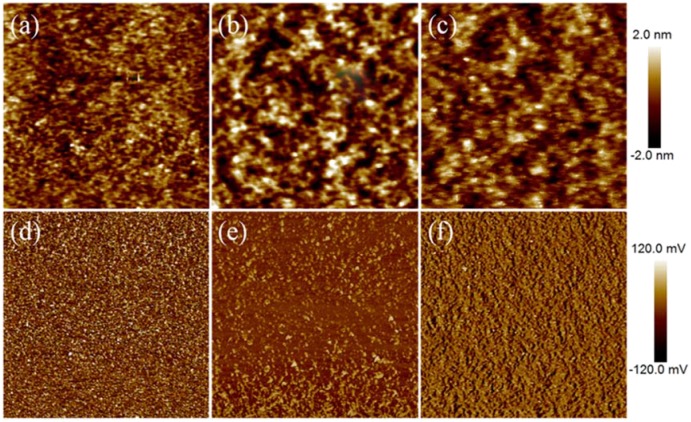
AFM height **(a–c)** and phase **(d–f)** images of thin films containing PBT-7-Th in blend with BTO-PDI **(a,d)**, BTT-PDI **(b,e)**, and BTSe-PDI **(c,f)**.

The crystallinity of the NFAs and nano phase separations of the corresponding blend films were characterized by using X-ray diffraction (XRD) and atomic force microscopy (AFM) techniques. The three XRD diffractograms of thin films containing the PTB7-Th/acceptor blends in Figure 7 show similar features with two diffraction peaks one around 5° and the other around 22.5° in 2θ. The two diffractions were corresponding to a lamellar packing distance of 17.7 Å and a π stacking distance of 3.9 Å from the PTB7-Th. Hence, the PDI derivatives remained predominantly amorphous in the thin films. The 3D fused propeller-like structure indeed largely prevented the generation of large crystals of PDI which was a source for the high performances. AFM images of thin films containing PTB7-Th/acceptor blends all showed homogeneously distributed sub-micrometer features in both height and phase images (Figure 8). The root mean square (RMS) of the height of thin films containing BTO-PDI, BTT-PDI, and BTSe-PDI were found to be 3.23, 2.65, and 4.55 nm, respectively. The smooth films also excluded the existence of micrometer sized crystals often found in conventional PDI based PSCs.

## Experimental

### Materials

All chemicals used for synthesis and device fabrications were purchased from Aladdin and Chron Chemicals without further purification. Compound **1a**, **1b**, **1c**, **2b**, and **3** were synthesized according to the previously reported procedures (Sonoda et al., 2001; Nicolas et al., 2004; Tsuji et al., 2014; Viswanath et al., 2014).

### Material Synthesis

#### Synthesis of Compound 2a

An *n*-butyllithium solution (1.62 mL, 4.04 mmol, 2.50 M in hexane) was added into a solution of compound **1a** (0.20 g, 1.01 mmol) in anhydrous tetrahydrofuran (30 mL) at 0°C and stirred for 1 h at room temperature under argon protection. After that, tributyltin chloride (1.90 mL, 4.10 mmol) was added into the mixture and stirred for 8 h at room temperature under argon protection. The obtained mixture was poured into deionized water, extracted by dichloromethane, dried with MgSO_4_ and filtered. The solvent was removed *via* rotary evaporation. The crude product was purified by column chromatography with petroleum ether as eluent to get the compound **2a** as a colorless oil (0.97 g, 89.56%). ^1^H NMR (400 MHz, CDCl_3_, δ/ppm): 7.32 (s, 3H), 1.68–1.60 (m, 18H), 1.41–1.34 (m, 18H), 1.24–1.17 (m, 18H), 0.95–0.91 (m, 27H). ^13^C NMR (100 MHz, CDCl_3_, δ/ppm): 137.10, 135.74, 132.72, 132.52, 130.77, 130.65, 130.54, 77.36, 77.24, 77.04, 76.72, 29.75, 29.15, 29.04, 28.94, 27.62, 27.33, 27.03, 13.72, 12.73, 12.65, 11.79, 10.96, 9.27. Anal. calcd for (C_48_H_84_O_3_Sn_3_) (%): C, 54.12; H, 7.95. Found (%): C, 53.72; H, 8.37.

#### Synthesis of Compound 2c

An *n*-butyllithium solution (6.40 mL, 10.20 mmol, 2.50 M in hexane) was added into a solution of compound **1c** (0.66 g, 1.70 mmol) in anhydrous tetrahydrofuran (50 mL) at 0°C and stirred for 2 h at room temperature under argon protection. After that, tributyltin chloride (3.0 mL, 11.10 mmol) was added into the mixture and stirred at room temperature for 12 h under argon protection. The obtained mixture was poured into deionized water, extracted by dichloromethane, dried with MgSO_4_ and filtered. The solvent was removed *via* rotary evaporation. The crude product was purified by column chromatography with petroleum ether as eluent to get the compound **2c** as a light-yellow oil (1.89 g, 87.40%). ^1^H NMR (400 MHz, CDCl_3_, δ/ppm): 7.86 (s, 3H), 1.69–1.60 (m, 18H), 1.39–1.32 (m, 18H), 1.25–1.17 (m, 18H), 0.95–0.89 (m, 27H). ^13^C NMR (100 MHz, CDCl_3_, δ/ppm): 141.16, 141.10, 138.67, 138.17, 137.94, 137.72, 136.20, 136.11, 135.21, 130.61, 130.55, 77.41, 77.30, 77.09, 76.78, 29.80, 29.23, 29.04, 28.94, 28.60, 27.56, 27.34, 27.22, 13.94, 13.78, 12.65, 12.57, 11.29. Anal. calcd for (C_48_H_84_Se_3_Sn_3_) (%): C, 45.97; H, 6.75. Found (%): C, 45.78; H, 6.81.

#### Synthesis of Compound 4a

A mixture of compound **3** (0.49 g, 0.68 mmol), compound **2a** (0.18 g, 0.17 mmol), degassed toluene (30 mL), and Pd(PPh_3_)_4_ (4% mol) was stirred for 20 h at 100°C under argon protection. The obtained mixture was poured into deionized water, extracted by dichloromethane, dried with MgSO_4_ and filtered. The solvent was removed *via* rotary evaporation. The crude product was purified by column chromatography with petroleum ether/dichloromethane (1:3) as eluent to get the compound **4a** as a dark red solid (0.124 g, 34.42%). ^1^H NMR (400 MHz, CDCl_3_, ppm): 8.89 (s, 3H), 8.71–8.65 (m, 12H), 8.34 (s, 3H), 8.03–8.01 (m, 3H), 7.49 (s, 3H), 5.23–5.11 (m, 6H), 2.27–2.17 (m, 12H), 1.84–1.82 (m, 12H), 1.33–1.25 (m, 48H), 0.86–0.75 (m, 36H). ^13^C NMR (100 MHz, CDCl_3_, ppm): 164.67, 163.54, 155.79, 146.89, 137.14, 135.02, 134.14, 133.50, 131.06, 129.25, 129.18, 128.92, 128.12, 128.03, 127.32, 126.25, 126.00, 124.15, 123.74, 123.45, 123.02, 119.69,111.72, 103.76, 77.27, 77.26, 77.05, 76.74, 54.76, 54.65, 32.94, 29.10, 22.62, 22.57, 14.05, 13.98. Anal. calcd for (C_138_H_138_N_6_O_15_) (%): C, 78.16; H, 6.56; N, 3.96. Found (%): C, 77.56; H, 6.96; N, 4.27.

#### Synthesis of Compound 4b

A mixture of compound **3** (0.5 g, 0.7 mmol), compound **2b** (0.19 g, 0.17 mmol), degassed toluene (30 mL), and Pd(PPh_3_)_4_ (4% mol) was stirred for 20 h at 100°C under argon protection. The obtained mixture was poured into deionized water, extracted by dichloromethane, dried with MgSO_4_ and filtered. The solvent was removed *via* rotary evaporation. The crude product was purified by column chromatography with petroleum ether/dichloromethane (1:3) as eluent to get the compound **4b** as a dark red solid (0.14 g, 38.00%). ^1^H NMR (400 MHz, CDCl_3_, δ/ppm): 8.85 (s, 3H), 8.74–8.67 (m, 12H), 8.57 (s, 3H), 8.45–8.39 (m, 3H), 7.94 (s, 3H), 5.20–5.16 (m, 6H), 2.34–2.22 (m, 12H), 1.90–1.78 (m, 12H), 1.39–1.16 (m, 48H), 0.94–0.79 (m, 36H). ^13^C NMR (100 MHz, CDCl_3_, δ/ppm): 164.49, 164.15, 163.26, 152.14, 145.69, 144.36, 143.47, 138.35, 137.24, 135.46, 134.13, 132.35, 129.01, 125.89, 123.45, 122.11, 119.67, 117.89, 55.83, 45.37, 42.70, 33.14, 32.03, 31.73, 30.47, 29.36, 26.47, 22.91, 21.79, 14.45, 13.34. Anal. calcd for (C_138_H_138_N_6_O_12_S_3_) (%): C, 76.42; H, 6.41; N, 3.87. Found (%): C, 76.36, H, 6.62, N, 3.92.

#### Synthesis of Compound 4c

A mixture of compound **3** (0.5 g, 0.7 mmol), compound **2c** (0.21 g, 0.17 mmol) degassed toluene (30 mL), and Pd(PPh_3_)_4_ (4% mol) was stirred for 20 h at 100°C under argon protection. The obtained mixture was poured into deionized water, extracted by dichloromethane, dried with MgSO_4_ and filtered. The solvent was removed *via* rotary evaporation. The crude product was purified by column chromatography with petroleum ether/dichloromethane (1:3) as eluent to get the compound **4c** as a red solid (0.14 g, 36.12%). ^1^H NMR (400 MHz, CDCl_3_, δ/ppm): 8.88–8.85 (m, 6H), 8.81–8.68 (m, 12H), 8.48 (s, 3H), 8.03 (s, 3H), 5.23–5.16 (m, 6H), 2.28–2.20 (m, 12H), 1.87–1.81 (m, 12H), 1.32–1.23 (m, 48H), 0.86–0.80 (m, 36H). ^13^C NMR (100 MHz, CDCl_3_, δ/ppm): 164.66, 163.53, 148.99, 137.82, 136.36, 134.90, 134.15, 133.45, 131.63, 129.13, 128.04, 127.35, 123.78, 123.06, 77.37, 77.26, 77.06, 76.74, 54.79, 54.72, 32.08, 29.72, 29.11, 22.63, 22.38, 14.07, 14.05. Anal. calcd for (C_138_H_138_N_6_O_12_Se_3_) (%): C, 71.77; H, 6.02; N, 3.64. Found (%): C, 70.56; H, 6.93; N, 3.87.

#### Synthesis of BTO-PDI

A mixture of compound **4a** (0.42 g, 0.20 mmol), degassed toluene (30 mL), anhydrous ferric chloride (1.62 g, 10 mmol), and nitromethane (5 mL) was stirred for 12 h at 100°C under argon protection. The obtained mixture was poured into deionized water, extracted by dichloromethane for three times, dried with MgSO_4_ and filtered. The solvent was removed *via* rotary evaporation. The crude product was purified by column chromatography with petroleum ether/dichloromethane (1:3) as eluent to get the compound **BTO-PDI** as a red solid (0.38 g, 90.52%). ^1^H NMR (400 MHz, CDCl_3_, δ/ppm): 11.64 (s, 3H), 10.81 (s, 3H), 9.49–9.36 (m, 6H), 9.29–9.05 (m, 6H), 5.82–5.39 (m, 6H), 2.49–2.33 (m, 12H), 2.10–2.00 (m, 12H), 1.42–1.34 (m, 48H), 0.86–0.82 (m, 36H). ^13^C NMR (100 MHz, CDCl_3_, δ/ppm): 166.38, 165.27, 163.93, 151.92, 150.81, 135.46, 133.90, 130.79, 129.68, 128.79, 127.45, 125.01, 123.67, 122.56, 120.56, 119.67, 109.21, 103.76, 55.38, 42.04, 32.69, 29.14, 23.35, 22.46, 14.01, 13.87. Anal. calcd for (C_138_H_132_N_6_O_15_) (%): C, 78.38; H, 6.29; N, 3.97. Found (%): C, 77.86; H, 7.26; N, 4.12. MS (MALDI-TOF-MS):[M]^+^: Calcd: 2113.97; Found: 2113.96.

#### Synthesis of BTT-PDT

A mixture of compound **4b** (0.43 g, 0.20 mmol), degassed toluene (30 mL), anhydrous ferric chloride (1.62 g, 10 mmol), and nitromethane (5 mL) was stirred for 12 h at 100°C under argon protection. The obtained mixture was poured into deionized water, extracted by dichloromethane, dried with MgSO_4_ and filtered. The solvent was removed *via* rotary evaporation. The crude product was purified by column chromatography with petroleum ether/dichloromethane (1:3) as eluent to get the compound **BTT-PDT** as a red solid (0.39 g, 89.14%). ^1^H NMR (400 MHz, CDCl_3_, δ/ppm): 10.95 (s, 3H), 9.61–9.56 (m, 6H), 9.48–9.47 (d, *J* = 7.9 Hz, 3H), 9.32–9.28 (m, 3H), 9.15–9.13 (m, 3H), 5.37–5.25 (m, 6H), 2.41–2.37 (m, 12H), 2.29–2.27 (m, 12H), 1.34–1.25 (m, 48H), 0.84–0.81 (m, 36H). ^13^C NMR (100 MHz, CDCl_3_, δ/ppm): 164.61, 164.13, 138.90, 137.98, 133.45, 131.51, 129.98, 129.13, 127.36, 127.11, 125.40, 125.27, 124.55, 124.06, 123.71, 123.21, 55.02, 54.67, 32.10, 29.71, 29.24, 29.07, 22.64, 22.49, 14.04, 14.01. Anal. calcd for (C_138_H_132_N_6_O_12_S_3_) (%): C, 76.64; H, 6.15; N, 3.89. Found (%): C, 75.56; H, 6.83; N, 4.37. MS (MALDI-TOF-MS):[M]^+^: Calcd: 2161.90; Found: 2161.89.

#### Synthesis of BTSe-PDT

A mixture of compound **4c** (0.46 g, 0.20 mmol), degassed toluene (30 mL), anhydrous ferric chloride (1.62 g, 10 mmol), and nitromethane (5 mL) was stirred for 12 h at 100°C under argon protection. The obtained mixture was poured into deionized water, extracted by dichloromethane, dried with MgSO_4_ and filtered. The solvent was removed *via* rotary evaporation. The crude product was purified by column chromatography with petroleum ether/dichloromethane (1:3) as eluent to get the compound **BTSe-PDT** as a red solid (0.41 g, 88.31%). ^1^H NMR (400 MHz, CDCl_3_, δ/ppm): 10.67 (s, 3H), 9.56–9.50 (m, 6H), 9.48–9.45 (d, 3H), 9.32–9.28 (m, 3H), 9.15–9.13 (m, 3H), 5.37–5.25 (m, 6H), 2.41–2.31 (m, 12H), 2.12–1.73 (m, 12H), 1.89–1.25 (m, 48H), 0.90–0.86 (m, 36H). ^13^C NMR (100 MHz, CDCl_3_, δ/ppm): 164.57, 163.97, 142.16, 141.15, 137.42, 135.34, 133.66, 133.11, 131.08, 130.07, 127.52, 127.16, 125.94, 124.72, 124.64, 124.09, 123.71, 122.84, 77.37, 77.25, 77.05, 76.73, 54.99, 32.16, 31.94, 29.72, 29.38, 29.14, 22.49, 22.48, 14.14, 14.03. Anal. calcd for (C_138_H_132_N_6_O_12_Se_3_) (%): C, 71.96; H, 5.78; N, 3.65. Found (%): C, 71.56; H, 5.83; N, 3.74. MS (MALDI-TOF-MS):[M]^+^: Calcd: 2303.74; Found: 2303.73.

### Materials Characterization and Methods

^1^H and ^13^C NMR spectra were recorded on a Bruker Avance-400 spectrometer with *d*-chloroform (CDCl_3_) or *d*-dimethyl sulfoxide [(CD_3_)_2_SO] as the solvents and tetramethylsilane (TMS) as internal standard. Thermalgravimetric analysis (TGA) and differential scanning calorimetry (DSC) analysis were carried out using a TA TGA Q500 and a Netzsch DSC 200, respectively, under N_2_ protective gas. The heating and cooling rates for thermal analysis were kept at 10°C/min. UV-vis absorption spectra were collected using a Persee TU1901 spectrometer. Cyclic voltammetry (CV) measurements were conducted using a CHI660 potentiostat/galvanostat electrochemical workstation at a scanning rate of 50 mV s^−1^. A platinum wire was used as the counter electrode and the reference electrode was Ag/AgCl with its energy level calibrated by a ferrocene/ferrocenium (Fc/Fc^+^) redox couple to be −4.34 eV. X-ray diffraction (XRD) was measured with a Philips X'pert X-ray diffractometer. Atomic force microscopy (AFM) images were recorded using a Bruker Innova Atomic Force Microscope in tapping mode.

### Device Fabrication and Measurements

PSC devices were fabricated with an inverted configuration (ITO/ZnO/active layer/MoO_3_/Al). Thirty nanometer thick ZnO layer was prepared with a Sol-Gel method on ITO glass substrates. The active layer of thickness around 100 nm containing PTB7-Th and the acceptor molecules (1:1.5 wt%) were spin coated onto the substrate from a chlorobenzene solution with total concentration of 25 mg/mL and 1% 1,8-diiodooctane as additive. MoO_3_ (10 nm) and Al (80 nm) layers were deposited onto the active layer *via* thermal evaporation. The device area was exactly fixed to 4.00 mm^2^

The *I*-*V* characteristics of the PSC devices were obtained by placing the PSCs under an AM 1.5G (100 mW cm^−2^) illumination created by a solar simulator (XES-70S1, SAN-EI, calibrated with a Konica Minolta AK-200 standard Si solar cell) and measuring using a Keithley 2400 Source Measure Unit.

The EQE curves were measured with a Newport QE Test Model during illumination with monochromatic light from a xenon lamp. The fabrication of PSC devices for *I*-*V* and EQE characteristic as well as the *I*-*V* measurements were conducted in a high purity argon filled glove box (<0.1 ppm O_2_ and H_2_O). EQE characteristics were performed in air on devices shortly removed from the glove box.

### Charge Transport Characterization

The charge carrier mobilities presented in this work were obtained using the space-charge limited conductivity (SCLC) method. Hole only (ITO/PEDOT:PSS/active layer/Mo_3_O/Au) and electron only (Al/active layer/Al) devices were fabricated similar to the PSC devices. The dark *J*-*V* current was collected by a Keithley 2400 Source Measure Unit and fitted to equation: $J\;=\;9\varepsilon_{0}\varepsilon_{r}\mu V^{2}/8L^{3}$, where *J* is the current density, *L* is the active layer thickness, μ is the charge transport mobility, ε_r_ and ε_0_ are the relative and free space (8.85 × 10^−12^ F m^−1^) permittivity, *V* is the internal voltage in device deduced from: *V* = *V*_*appl*_ − *V*_*bi*_ − *V*_*a*_, where *V*_appl_ is the applied voltage, *V*_bi_ is the built-in voltage and *V*_a_ is the voltage drop.

## Conclusions

In this work, we designed and synthesized three fused propeller-like PDI derivatives namely BTO-PDI, BTT-PDI, and BTSe-PDI by introduce an additional chalcogen (oxygen, sulfur, and selenium, respectively) linkage to our previously reported Ph-PDI molecule to form rigid fused linkages. The fused PDI derivatives show flatter molecular conformation and more delocalized HOMO with deeper HOMO energy levels. The larger band gaps provide blue shifted absorption which was beneficial for the complimentary absorption incorporated with the donor polymer PTB7-Th. The propeller-like structure largely prevented the formation of large PDI crystals. In comparison between the three NFAs introduced in this work, heavier chalcogen linkage reduced the LOMO energy level and the *V*_oc_ of the devices. Highest PSC performances was found with the BTT-PDI which combines high absorption with high electron transport mobility. Our work demonstrated the great potential of PDI derivatives for PSC application and explored the influences of linkage type on the fused PDI derivatives which provide a useful tuning knob for molecular design of NFAs.

## Data Availability Statement

The original contributions presented in the study are included in the article/supplementary materials, further inquiries can be directed to the corresponding author/s.

## Author Contributions

YL: design and synthesis of the materials. YG: synthesis of the materials. YC: characterization of the materials. XX: fabrication and characterization of the devices. LY: analysis of the results and organization of the manuscript. QP: affording the idea, directing this work and organization of the manuscript.

## Conflict of Interest

The authors declare that the research was conducted in the absence of any commercial or financial relationships that could be construed as a potential conflict of interest.
